# A bioinspired synthetic soft hydrogel for the treatment of dry eye

**DOI:** 10.1002/btm2.10227

**Published:** 2021-06-05

**Authors:** Yu Yu, Derek Wai Yee Chow, Chi Ming Laurence Lau, Guanqun Zhou, Woojin Back, Jing Xu, Sean Carim, Ying Chau

**Affiliations:** ^1^ Chemical and Biological Engineering Hong Kong University of Science and Technology Hong Kong China; ^2^ Pleryon Therapeutics Limited Shenzhen China; ^3^ Veterinary Specialty Hospital Hong Kong China; ^4^ The Hong Kong University of Science and Technology Shenzhen Research Institute Shenzhen China

**Keywords:** Blob model, dry eye, hydrogel, soft hydrogel

## Abstract

Natural soft hydrogels are unique elastic soft materials utilized by living organisms for protecting delicate tissues. Under a theoretical framework derived from the Blob model, we chemically crosslinked high molecular weight hyaluronic acid at a concentration close to its overlap concentration (c*), and created synthetic soft hydrogels that exhibited unique rheological properties similar to a natural soft hydrogel: being dominantly elastic under low shear stress while being viscous when the stress is above a small threshold. We explored a potential application of the hyaluronic acid‐based soft hydrogel as a long‐acting ocular surface lubricant and evaluated its therapeutic effects for dry eye. The soft hydrogel was found to be biocompatible after topical instillation on experimental animals' and companion dogs' eyes. In a canine clinical study, twice‐a‐day ocular instillation of the soft hydrogel in combination with cyclosporine for 1 month improved the clinical signs in more than 65% of dog patients previously unresponsive to cyclosporine treatment.

## 1 INTRODUCTION

Soft hydrogel is a liquid‐like solid^1^ commonly found in animals and plants as a soft protectant. These soft hydrogels have extraordinary properties that distinguish them from other materials: the mechanical strength of soft hydrogel is so weak that it could not stand its own weight; the gels are elastic at rest but flowable; and they could retain the elasticity after a history of flow. For example, the mucus in human respiratory track is a soft hydrogel that functions as a protective layer.^2^ When the mucus is at rest, it coats and protects the delicate airway surface. During coughing, it easily flows without damaging the underlying tissue. When coughing stops, the protective function is restored. Changes in the mechanical property of this soft hydrogel are associated with lung diseases.^2^, ^3^, ^4^ Other examples of the soft hydrogel include the vitreous humor in the eye^5^ and egg white,^6^ where soft hydrogels provide mechanical support and protection of the most delicate tissues in living organisms including the retina and the embryo.

In rheological terms, soft hydrogel is characterized by an exceptionally low storage modulus (*G*′) value (on the order of 0.01–1 Pa) at low shear frequency oscillation while its value is still higher than the loss modulus (*G*″). The gel deforms elastically under a very low stress (on the order of 1 Pa or lower), but deforms viscously when the stress is above a small threshold. Thus, the soft hydrogel is not a viscoelastic polymer solution (or a “structured liquid”^1^), which has *G*′ higher than *G*″ only at high shear frequency oscillation, and deforms viscously regardless of the applied stress.

Since nature has designed soft hydrogel as a protective barrier for various biological tissues, a synthetic soft hydrogel would be of great interest and potential in medicine as a protectant for delicate tissues. One potential is in the treatment of dry eye, which is one of the most common eye diseases affecting 20–30% population globally.^7^ Corneal epithelium damage and lid wiper epitheliopathy caused by precorneal friction are one of the core mechanism of dry eye.^8^ However, previous effort in making hydrogel for dry eye treatment are not optimum. For example, contact lens has been suggested as a drug delivery device for dry eye treatment^9^; however, contact lens wear could induce dry eye^10^ because of the high modulus of the material.^11^ Synthetic soft hydrogels have also been developed.^12^, ^13^, ^14^ However, the modulus is on the order of 10–100 Pa, which is too high compares to natural soft hydrogel and not applicable for precorneal application. Thiolated polymers based hydrogels have been used for dry eye treatment; however, the modulus was either too high^12^ or the difference in *G*′ and *G*″ is not significant,^15^ which indicated that material is more fluidic than elastic.

Here, we present a theoretical framework and material platform for making synthetic soft hydrogels, and characterize the unique rheological properties of soft hydrogels so prepared. The superior effect of the synthetic soft hydrogel for dry eye treatment was demonstrated to illustrate for its biomedical potential.

### 1.1 Theoretical consideration

**FIGURE 1:**
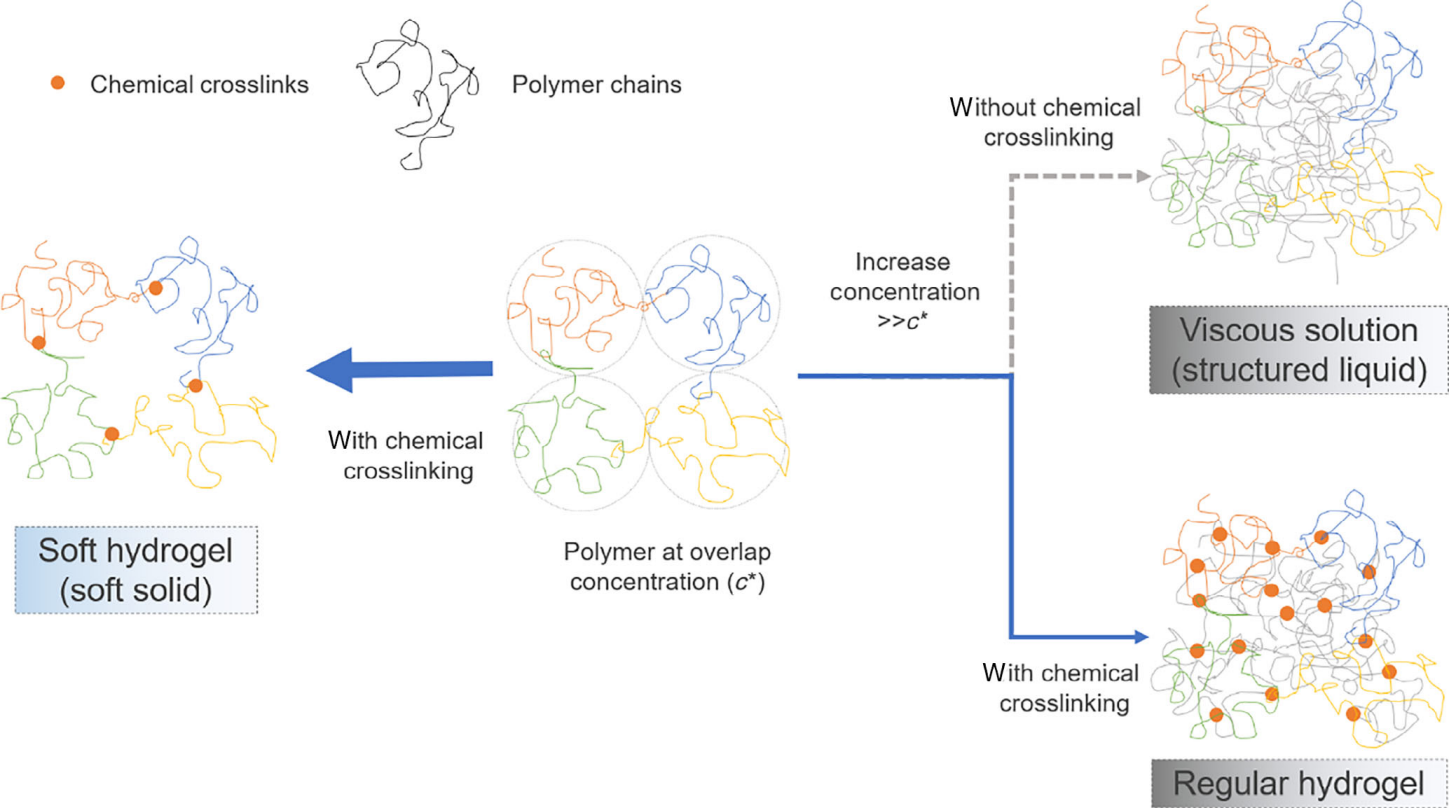
Illustration of the difference between a soft hydrogel, a viscous solution, and a conventional hydrogel. The orange dots represent crosslinks, the threads of different color represent different polymer chains. Center: a polymer solution where the polymer concentration is at the overlap concentration (c*) where the polymer chains are just dense enough to touch each other. The dotted circles represent the average space (Blob size) one polymer occupied. To the right: with increasing polymer concentration above c*, polymer chains become increasingly entangled and the solution turns viscous.^16^ Conventional hydrogels are made by crosslinking the polymers are such condition. To the left: the chains of a polymer solution at a concentration about c* are crosslinked to form a hydrogel of the lowest possible crosslinking density for such polymer

Viscous solution (“structured liquid”) that are being widely used in biomedical application are usually composed of polymers dissolved at high concentration (or at the semi‐dilute regime, Figure 1). The polymer chains of viscous solution are only weakly associated at the entanglement points, and the chain slips and the bulk material deforms viscously when a stress is applied. In contrast, the natural soft hydrogels are composed of polymers that are chemically or physically crosslinked at an extremely low concentration and crosslinking density.^17^ We are inspired by this natural phenomenon and reasoned that a synthetic soft hydrogel can be made when the polymer is crosslinked at a very low crosslinking density (Figure 1).

According to the Blob model, for hydrogel made by crosslinking of polymers, the maximum density of crosslinks is determined by the density of polymer entanglement points, which is determined by the concentration of a given polymer.^18^ For this reason, the lowest possible number of crosslinks appears at the threshold concentration when the polymer chains start to entangle, that is, near the overlap concentration c* for a given polymer solution (Figure 1). One can roughly relate c* to the properties of the polymer by Equations (1) and (2)^16^:

(1) $$c^{*}=\frac{M}{N_{A}\times \frac{4\pi }{3}{R_{g}}^{3}},$$

(2) $$c^{*}=\frac{1}{\left[\eta \right]},$$

where *M* is the molecular weight of the polymer, *N*_*A*_ is the Avogadro's number, *R*_*g*_ is the radius of gyration of a polymer chain, and [η] is the intrinsic viscosity of the polymer. And since [η] can be measured experimentally by simple techniques and has been tabulated for many polymers, c* can be easily predicted.^18^

From this analysis, it is clear that given the same molecular weight, polymers having a more extended configuration will achieve lower c*. For polymer of the same species, lower c* can be achieved by higher molecular weight. A chemical crosslink is preferred over a physical crosslink because the physical crosslink could be unstable under extremely low crosslinking density. In comparison, a conventional hydrogel usually composes of crosslinked polymers at semidilute concentration that is much higher than the overlap concentration (*c* ≫ c*, Figure 1).

## 2 RESULTS

### 2.1 Making synthetic soft hydrogel from chemically crosslinked hyaluronic acid

Based on the theoretical consideration, we synthesized soft hydrogels by chemically crosslinking a polymer at a concentration closed to c*. We identified hyaluronic acid (HA) to be a suitable material for forming soft hydrogel, both as an illustration of the principle and for the application in dry eye treatment. HA is a naturally existing polymer that is found in soft hydrogels in the body^19^, ^20^ and on ocular surface.^21^, ^22^, ^23^ The configuration of HA in aqueous solution is one of the most extended among polymers that have also been used for biomedical applications (Table S1).

HAs of molecular weight ranging from 120 kDa to 2.6 MDa were modified with vinylsulfone (VS) and thiol (SH) groups with a certain degree of modification (DM) by following our reported modification methods.^18^, ^24^, ^25^, ^26^ The modified HA‐VS and HA‐SH were tested for the ability to form soft hydrogels at concentrations above and below c*.

As expected, gelation happened only when polymer concentration was above c* (Table 1). The storage modulus (*G*′) of the gel is dependent on the concentration of the polymer. Since polymers with higher MW have lower c*, gels formed by HA of 2.6 MDa near c* give the lowest G' (Table 1). The storage modulus (*G*′), despite having a very small value on the order of millipascal, is higher than the loss modulus (*G*″) at low frequency of oscillation, indicating that the solid property is dominant over the liquid property (Table 1 and Figure S4), which is characteristic of a soft hydrogel.

**TABLE 1:** Gel formation capability and rheological properties of hyaluronic acid hydrogels varying in molecular weight and concentration

MW (Da)	Concentration (mg/mL)	*c*/c*	*G*′ (Pa)	*G*″ (Pa)
120 k	3.96	1.1	40.52 ± 0.39	9.03 ± 1.98
	1.65	0.5	No gel formation	
670 k	1.80	2.0	17.44 ± 0.49	5.33 ± 0.33
	1.08	1.2	4.70 ± 0.40	1.55 ± 0.12
	0.45	0.5	No gel formation	
2.6 M	1.20	3.6	3.8 ± 0.09	0.13 ± 0.01
	0.80	2.4	0.87 ± 0.03	0.07 ± 0.01
	0.50	1.5	0.12 ± 0.01	0.03 ± 0.01
	0.27	0.8	No gel formation	

*Notes*: The storage modulus (*G*′) and loss modulus (*G*″) were measured by rheometer at 5% strain and 5 rad/s. c* is the overlap concentration of the polymer estimated from [*η*], *c* is the concentration of the given polymer precursors. The degree of modification was 28 ± 1% for all polymers. Measurements were triplicated for each formulation (mean ± SD).

### 2.2 Elastic (solid) behavior of soft hydrogel

**FIGURE 2:**
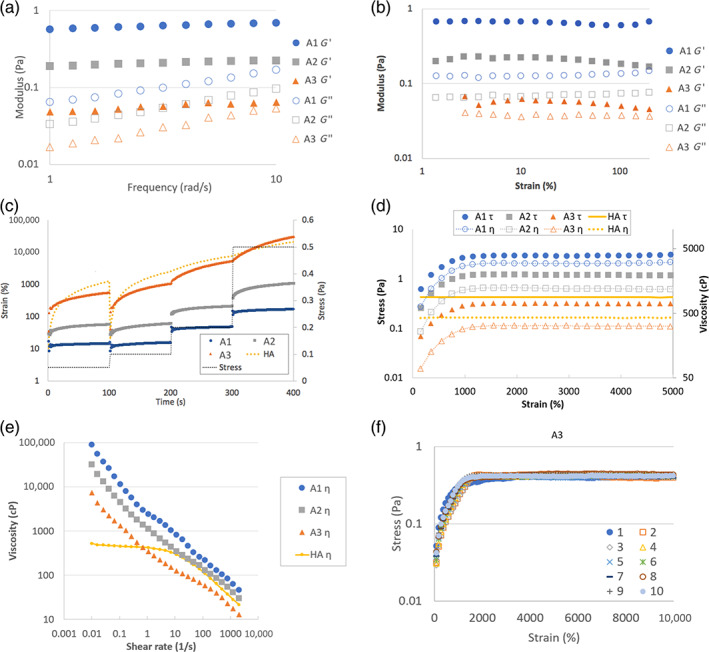
Rheological properties of soft hydrogels A1, A2, and A3. (a and b) Oscillatory test for soft hydrogels. The strain was 5% in the frequency sweep test (a) and the frequency was 5 rad/s in the strain sweep test (b). (c) The change of strain of the soft hydrogels and the HA solution in a step‐stress test. The stress was 0.05, 0.1, 0.2, and 0.5 Pa. (d) The stress and viscosity of the soft hydrogels and the HA solution (1.5 MDa HA at 5 mg/mL) in a continuous shear test at a constant shear rate of 1/s. (e) Viscosity change of the soft hydrogels and HA solution in continuous shear test with variable shear rate from 0.01/s to 2000/s. (f) The stress response to continuous shear test at 1/s shear rate were measured for 10 repeated times of formulation A3. Each symbol represents one repeat

Soft hydrogels with *G*′ ranging from 0.05 to 0.5 Pa (prepared using 2.6 M HA as detailed in Table 2) were further investigated for their elastic behavior. All hydrogels had higher *G*′ compared to *G*″ at the strain and frequency range tested (Figure 2a,b). The *G*′ and *G*″ values were about 0.7 and 0.1 Pa, 0.2 and 0.05 Pa, 0.05 and 0.03 Pa in formulation A1, A2, and A3, respectively. The elasticity was also demonstrated by the step‐stress test (creep analysis) performed at 0.05, 0.1, 0.2, and 0.5 Pa (Figure 2c). For gel A1, the strain remained almost constant over time at each stress level, indicating the material responds by deformation rather than flow. Gel A2 behaved similarly to A1 except that a larger strain was found for each stress, and a more significant creep at the larger stress. Gel A3, despite its very low *G*′ (~0.05 Pa), behaved as a viscoelastic solid at the lower two stress levels. Another proof of elasticity was shown by the repeats of small magnitude oscillation in strain at the beginning of the test (Figure S5). This phenomenon, termed inertio‐elastic ringing,^27^ was a strong indication for elasticity and was observed in all the soft hydrogel tested but not in a viscous solution.

**TABLE 2:** Formulations of hydrogel

Code	DM of HA‐VS (%)	DM of HA‐SH (%)	HA‐VS concentration (mg/mL)	HA‐SH concentration (mg/mL)	Additional treatment
A1	15	10	0.6	0.3	NA
A2	15	10	0.6	0.3	Autoclaved
A3	15	10	0.6	0.2	NA

### 2.3 Viscous (liquid) behavior of soft hydrogel

We next evaluated if the synthetic soft hydrogel could flow (viscous deformation) when the stress was above a threshold level. We first measured how the material would respond to a continuous shear deformation at a constant shear rate of 1/s (Figure 2d). The stress response (and thus the viscosity) was constant for HA solution, as expected of a viscous solution. The soft hydrogels, on the other hand, showed an increase in stress and viscosity up to about 1500% strain. This indicates that elastic deformation is a dominant phenomenon for these gels at strain lower than 1500%. Above this strain level, the stress and viscosity became relatively constant, indicating that the soft hydrogels flew like a viscous solution. The yield stress was about 3.0, 1.5, and 0.3 Pa for gel A1, A2, and A3, respectively. We then evaluated the flow behavior of the hydrogels at shear rates from 0.01/s to 2000/s (Figure 2e). When the shear rate was increased to above 1000/s, the viscosity of the material became similar to or lower than a viscous solution. At 2000/s, the viscosity was 46, 30, and 13 cP for hydrogel for the three formulations, respectively, and 21.6 cP for the HA solution.

### 2.4 Behavior of soft hydrogel after large deformation

The previous sections showed that the synthetic soft hydrogels were able to undergo both elastic and viscous deformation, we went on to evaluate if the elasticity could be retained after the viscous deformation.

The stress response of the soft hydrogel was measured in a continuous shear test at a constant shear rate of 1/s for 100 s (to attain 10,000% strain). The test was repeated 10 times with a 10‐s‐pause between tests. The total strain applied to the hydrogels after 10 repeats was 100,000%. Surprisingly, for all the three hydrogels tested, the stress response curves were almost identical for all the repeats: there was an initial elastic deformation until about 1500% strain, followed by a viscous deformation at almost identical stress value (Figure 2f and Figure S6). This experiment showed that the soft hydrogels were able to repeatedly switch from elastic to viscous deformation.

### 2.5 In vivo study in laboratory animals

**FIGURE 3:**
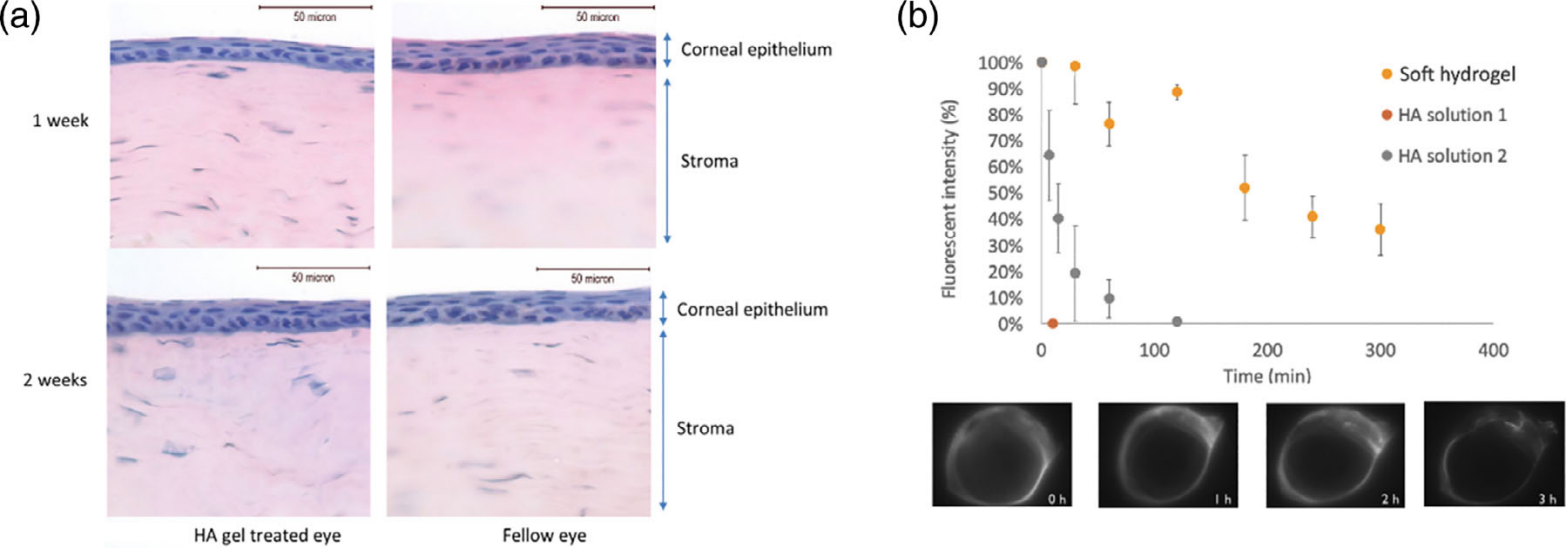
In vivo rabbit study. (a) Representative H&E staining showing the histology of cornea treated with HA gel and saline 1 and 2 weeks post instillation. (b) Precorneal retention of fluorescently labeled soft hydrogel, an HA‐VS solution and HA polymer solution of high viscosity. HA solution 1 contains fluorescently labeled 2.6 MDa HA‐VS of 15% DM at 0.45 mg/mL. HA solution 2 contains of fluorescently labeled 1.5 MDa HA at 5 mg/mL. Measurements were triplicated for each formulation (mean ± SD)

The biocompatibility of the hydrogel was evaluated in rats and rabbits for single and repeated instillations (Figure S7). The eyes were inspected for changes in gross appearance and signs of infection and discomfort, such as swelling, hyperemia, changes in corneal clarity and mucoid discharge. In all the studies, the cornea remained clear and the conjunctiva showed no signs of inflammation. Corneal staining of rabbit eyes was graded 0 immediately and 2 weeks post instillation, suggesting that the soft gels did not cause any damages to the corneal epithelium. Histological examination of the rabbit cornea did not find any gross change in the corneal epithelium (Figure 3a). The epithelium thickness and integrity were similar in hydrogel and saline treated eyes. Also, no sign of inflammatory response was observed.

To evaluate whether the soft hydrogel can prolong precorneal residence, the gel was made by crosslinking fluorescent‐labled HA‐VS with HA‐S and instilled on the ocular surface of anesthetized rabbits. The result was compared with fluorescent‐labled HA‐VS solution (the precursor of hydrogel) and a viscous formulation of fluorescent‐labled HA (Figure 3c). In the absence of chemical crosslinking, the fluorescence intensity decreased to the background level within 3 min. The viscous formulation of HA extended the half‐life to about 10 min. On the other hand, the soft gel stayed on the ocular surface for at least 5 h. At the end of the experiment, the fluorescence intensity remained at the level of about 40% of the initial value.

### 2.6 Veterinary clinical study

We went on to evaluate if the prolonged retention of the HA soft gel on the ocular surface would be beneficial to the treatment of companion dogs with dry eye.

The biocompatibility of the soft hydrogel (Formulation A3, Table 2) was confirmed in five healthy dogs. We found that the gel was well tolerated by the dogs and well received by the dog owners. The soft gel can be instilled easily from a unidose eye drop container like conventional eye drops (Figure S8).

**FIGURE 4:**
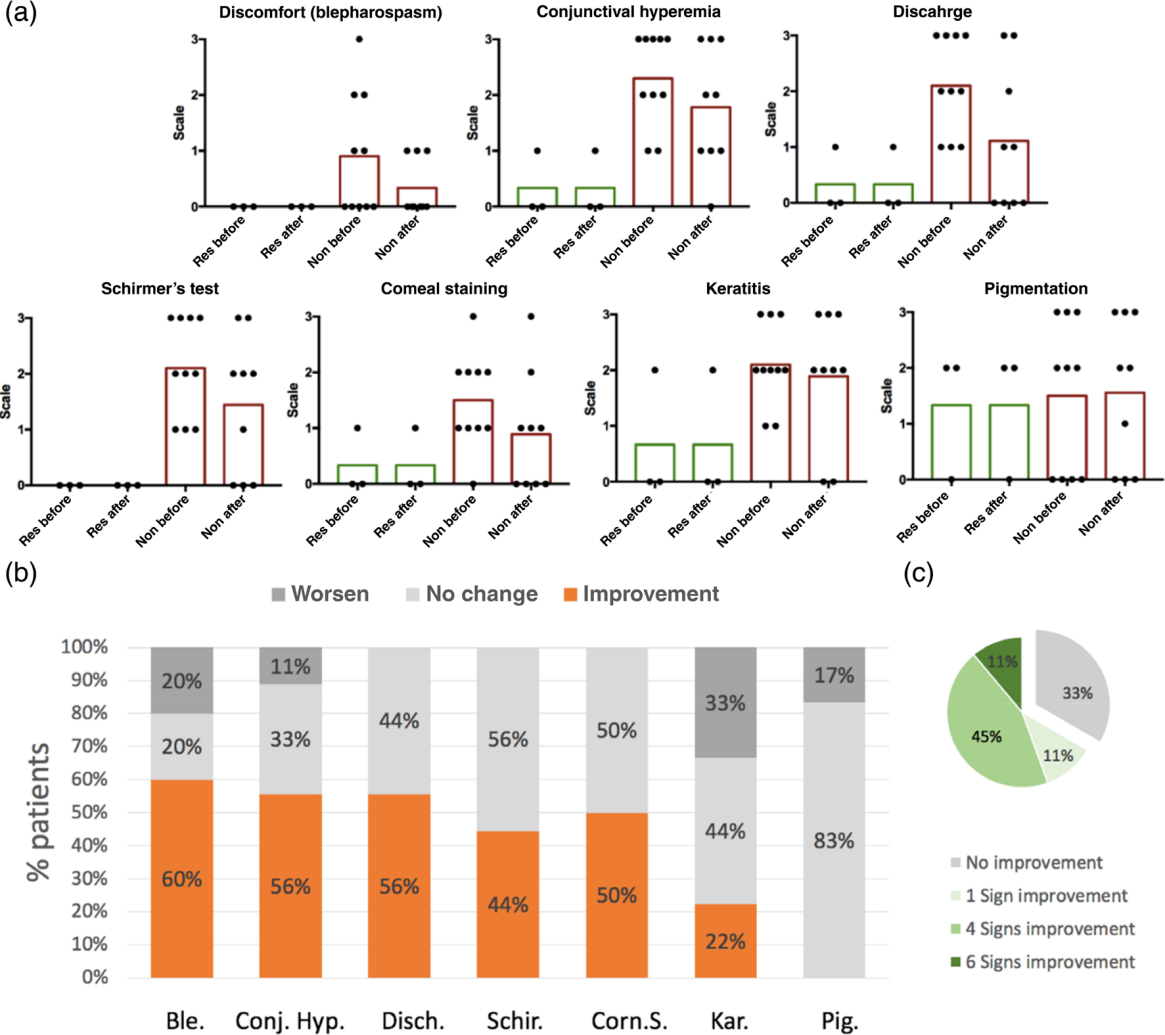
Results from canine study. (a) Grading of clinical signs before and after hydrogel treatments. Green bar: mean grading value of cyclosporine‐responsive group. Red bar: mean grading value of cyclosporine nonresponsive group. Before: before treatment; After: 1 month after soft hydrogel treatment. (b) Percentage of patients' different responses to 1‐month soft hydrogel treatments. Only patients' having a particular sign with grade > 0 before the treatment were included. The seven clinical signs are blephorospasm (Ble.), conjunctiva hyperemia (Conj. Hyp.), discharge (Disch.), Schirmer's test (Schir.), corneal staining (Corn. S.), keratitis (Kar.), and pigmentation (Pig.). (c) Percentage of cyclosporine nonresponsive dog patients having different number of sign improvement

To evaluate the efficacy of the gel in dry eye population, dog patients diagnosed with dry eye disease and had been on conventional therapy (BID 1% cyclosporine and 4–6 times per day artificial tear) for over 12 months were enrolled in the study. Based on the clinical outcome of the previous cyclosporine treatment, patients were divided into cyclosporine‐responsive group (Schirmer's test graded at 0 at the time of enrollment) and cyclosporine‐nonresponsive group (Schirmer's test graded at 1–3). The dry eye clinical signs of the patients were graded before and after treatment based on the scale listed in Table 3. The grading scale is similar to the scale used for human diagnosis,^28^ wherein a higher number indicates higher severity of the disease. The results of the clinical study are summarized in Figure 4a. In this figure, each dot represents the grading of one dog eye. In this study, almost all dogs have unilateral dry eye. For cyclosporine responsive dogs, the new treatment was able to maintain the therapeutic effect of the previous treatment, despite the reduction in instillation frequency. For cyclosporine nonresponsive dogs, the majority experienced a reduction in clinical signs after the treatment. In five clinical signs, 44–60% of the dog showed at least one grade of improvement (Figure 4b). The reduction in the grading of blepharitis, conjunctival hyperemia and discharge, as well as the increase in tear production as measured by Schirmer's test, indicated the overall health of the ocular surface had improved. The reduction in corneal staining indicated an improvement in corneal health. For cyclosporine nonresponsive dogs, 66% of the patients had improvement in at least one clinical sign and 56% in at least four clinical signs (Figure 4c).

**TABLE 3:** Clinical signs evaluated in the clinical study and the grading scale

Clinical sign and symptom				
Scale	0	1	2	3
Discomfort (blepharospasms)	None	Seldom	Intermittent	Constant
Conjunctival hyperemia	None	Mild	Moderate	Severe
Cornea (keratitis)	None	Limbal	50% of cornea	Complete
Cornea (pigmentation)	None	Peripheral	50% of cornea	Complete
Discharge	None	Thickened but not discolored	Purulent	Purulent with dry discharge adhered to the cornea
Schirmer's test	~15	10–15	5–10	<5
Corneal staining	None to mild	Variable	Marked central	Severe punctate erosions

## 3 DISCUSSION

### 3.1 Formation of HA‐based soft hydrogel

By forming hydrogel from large size polymer (HA of 2.6 MDa) at the concentration closed to c*, soft hydrogel can be synthesized. The formed soft hydrogel is elastic at low shear stress (Figure 2a–c) and flowable in the presence of a relatively low yield stress (Figure 2d and e).

The soft hydrogel has very unique physical appearance. For example, when we shake the bottle containing the soft hydrogel, the hydrogel appears to be a nonviscous solution (see Movie S1). However, when we put a fluorescently labeled soft hydrogel in water, it will not be dissolved for days and move along with water when we tilted the tube (Movie S2). In contrast, a viscous solution of polymer would have a “sticky” appearance and it will be quickly dissolved when mixed with water.

Another surprisingly feature of the soft hydrogel was the ability to retain the elasticity after a history of repeated viscous deformation (Figure 2f). The study of the mechanism for this behavior is beyond the scope of this study. We hypothesize that the soft hydrogel may consist of a certain percentage of non‐covalent entanglements, which may be torn apart more readily and yet quickly recovered afterwards. The covalent bonds were only minimally disrupted upon shearing, and thus the gel retains the elasticity. An alternative explanation is that although the hydrogel's covalent bonds were broken by the large deformation, the hydrogel was split into a few bulk pieces. Because the gel has low modulus, these pieces may deform to fit tightly into each other and behave as a bulk elastic material. The non‐covalent interactions of the polymer chains may further enhance such “healing” effect. Regardless of the mechanism, our result clearly demonstrates that the material can recover from very large deformation.

### 3.2 Design criteria of soft hydrogel for dry eye and its precorneal retention

Currently, topical application of artificial tears drops is the first line treatment of dry eye.^29^ However, artificial tears are cleared rapidly from the corneal surface (typically within minutes),^30^, ^31^ and frequent instillations as many as multiple times per hour^32^ are needed while the therapeutic effect is still suboptimal. Previous efforts to prolong the precorneal residence time by increasing the viscosity of artificial tear have been ineffective because the solution is quickly diluted and cleared from the lacrimal system.^33^, ^34^ The higher viscosity also causes discomfort to the patients and lowers patient compliance.

To design a proper soft hydrogel for dry eye therapy, three aspects should be considered. First, the gel should be able to behave elastically under the stress exerted at the puncta. Precorneal fluid is cleared from flowing through the punta to the lacrimal drainage system.^35^, ^36^, ^37^, ^38^ The stress produced by the suction at the puncta is estimated to be around 0.04–0.07 Pa (Supporting Information and Figure S9). A soft hydrogel that behaves elastically under this stress is expected to deform at rather than flow through the puncta, leading to a longer residence time on the ocular surface compared to conventional eye drops. Second, the viscosity of the hydrogel should be similar to the conventional eye drop at a shear rate of about 1200/s (the estimated shear rate during blinking^39^). The human eye is very sensitive to the viscosity of any materials on the ocular surface, and an increase in viscosity during blinking may cause higher shear stress to the underlying corneal epithelium.^40^ Thus, a relatively low shear viscosity at a high shear rate is important for patient comfort and long‐term biocompatibility. Lastly, the rheological properties should be recoverable after large deformation. This property means that the soft hydrogel can maintain the benefits from its elasticity after blinking. Another practical benefit is that the soft gel can be extruded through a conventional eye drop applicator readily without compromising its unique rheological properties (Figure S10 and Movie S3).

For these reasons, soft hydrogels with *G*′ in the range of 0.2–0.05 Pa are suitable candidates to be evaluated for dry eye treatment. And our result shows that the precorneal retention of soft hydrogel was over an order of magnitude longer compares to viscous solution (Figure 3c).

### 3.3 Treatment of dry eye disease in dog patients

Dry eye is a multifactorial disease and most of the animal models available are not representative to the human disease.^8^ Spontaneous dry eye disease in companion canine on the other hand, has been found to be clinically and immunopathologically similar to dry eye in human.^41^ For this reason, we conducted the efficacy study of the soft hydrogel on a heterogenous population of companion dog patients of Veterinary Specialty Hospital (VSH) in Hong Kong, to enhance to translational perspective of this study.

Our results demonstrated that twice per day instillation of soft hydrogel in combination of cyclosporin for 1 month was able to improve the clinical signs of more than 65% of patients that were unresponsive to previous cyclosporine treatment. Although cyclosporine is often prescribed for human dry eye, only about 12% of the patients are responsive to the therapy.^42^ Our results suggest that HA based soft hydrogel may be a new treatment option beneficial to dry eye patients unresponsive to the current cyclosporine regime. The significant improvement of clinical signs in a high percentage of patients suggests that the prolonged retention of HA soft hydrogel on the ocular surface is a promising strategy to improve the health of the ocular surface.

A limitation of the study is that the clinical study is performed as an open label, single‐arm study without a regular treatment group. Although the “switch” design is intended to evaluate therapeutic effect of the conventional and soft gel therapy for the same dog, comparing the soft gel treatment group with a second arm of conventional therapy would be included in a future study.

## 4 MATERIALS AND METHODS

### 4.1 Preparation of HA‐VS and HA‐SH

HAs were modified with pedant VS as described by previously.^24^ Briefly, HA was dissolved in double deionized water (DDI water). Five molar NaOH was added drop wise to the polymer solution to a final concentration at 0.1 M. Divinylsulfone (DVS) was added instantly with vigorous mixing. The reaction was stopped by adding 6 M HCl. The polymers were purified by membrane separation using dialysis bag or tangential flow filtration (TFF) against DDI water (about pH 5.5). The purified polymer was stored as a solution at 4°C. To determine the degree of modification (DM), HA‐VS was freeze dried and measured by ^1^HNMR. The detail experiment conditions can be found in Supporting Information (Figure S2).

HAs were modified with pedant SH group as described by Yu and Chau.^18^ Briefly, HA was first modified to HA‐VS. The HA‐VS was converted to HA‐SH by reacting with dithiothreitol (DTT) in nitrogen environment. The reaction was allowed for 25 min and stopped by adding 1 M HCl to reduce the pH to 3.5–4.5. The polymers were purified by membrane separation using dialysis bag or tangential flow filtration against DDI water of pH 4 adjusted by HCl. The purified polymer was stored as a solution at 4°C. The degree of modification (DM) was determined by Ellmans' assay.

### 4.2 Hydrogel formation

The concentration of HA‐VS and HA‐SH was first determined from measurement of freeze dried weight or by using a modified cetyltrimethy‐lammonium bromide (CTAB) assay.^43^ For the CTAB assay, CTAB was dissolved in 2% NaOH solution. HA‐VS or HA‐SH solution was diluted with 0.1 M phosphate buffer of pH 7.4. The assay was performed in 96 well plate. For each well, 100 μL sample was mixed with 100 μL CTAB solution. After mixing, the plate was immediately transferred to a 37 °C oven and incubated for 10 min, and was immediately read in a plate reader for absorbance at 595 nm. The concentration was determined by comparing the absorbance with unreacted HA processed with the same method.

HA‐VS and HA‐SH of known concentration was adjusted to pH 7.4 by the addition of 0.5 M PB. The osmolality was then adjusted using 25% NaCl. The polymers were then mixed at various target volume ratio and mass ratio, and adjusted to the target concentration by adding phosphate buffered saline (PBS). The polymers were incubated at 37°C for 24 h for hydrogel formation.

A list of hydrogel formed by 2.6 MDa HA is summarized in Table 2. Formulation A2 were formulation A1 autoclaved at 121°C for 20 min. The higher DM and mass ratio of VS over SH in these formulations was to avoid shrinking of hydrogel during long‐term storage.^44^

### 4.3 Mechanical properties measurement of the hydrogel

For hydrogel having *G*′ > 1 Pa, the mechanical properties were measured by ARES Rheometer (TA Instruments, New Castle, DE) on a 50 mm plate fixture.

For hydrogel having *G*′ < 1, the mechanical properties were measured using MCR 102 rheometers (Anton Paar GmbH, Graz, Austria). The hydrogel forming polymers were mixed and incubating at 37°C for 24 h in a centrifuge tube. The hydrogel was then loaded onto the lower plate of a 60 mm cone‐plate fixture (CP60‐1/T1), and the mechanical properties were measured.

### 4.4 Precorneal retention of hydrogel

#### 4.4.1 Fluorescent tagging of the hydrogel

The hydrogel was made fluorescent by tagging a fluorescent dye on the HA‐VS polymer. A thiol modified fluorescein was used. The dye was made by reacting amino‐fluorescein with N‐acetyl‐S‐trityl‐l‐cysteine (GLS Biochem Ltd, Shanghai, China) in DMF, catalyzed by HOBT/HBTU. To conjugate the dye to HA‐VS, HA‐VS solution was adjusted to pH 8.8 using tris buffer and react with the dye in nitrogen environment for 12 h. The polymer was purified by dialysis and the storage condition was the same as for HA‐VS. Then, the fluorescent‐labeled HA‐VS was mixed with HA‐SH and hydrogels were formed after incubation for 24 h at 37 °C.

#### 4.4.2 Animal experiment and animal preparation

Animal experiments were approved by the Animal and Plant Care Facility at the Hong Kong University of Science and Technology (15–143 in DH/HA & P/8/2/2 Pt. 8). 1.5‐year‐old NZW rabbits were used for in vivo precorneal retention study. The rabbits were first anesthetized by intramuscular injection (0.8 mL/kg body weight) of a mixture of 2% xylazine/10% ketamine at a ratio of 1:1. When the experiment was conducted longer than 1 h, the rabbit was further anesthetized with injection of the xylazine/ketamine mixture at 0.6 mL/kg body weight every hour.

#### 4.4.3 Imaging the precorneal clearance of hydrogels and polymers

An imaging system comprises an excitation module and an emission detection module were assembled in house (Supporting Information and Scheme S1).

Retention of fluorescein labeled hydrogel was monitored by imaging the ocular surface at predetermined illumination intensities and time points. Experiments were conducted in a dark room. Before hydrogel or solution was instilled, the background fluorescence of each eye was measured. Afterwards, 50 μL of hydrogel was instilled onto the center of the cornea. After installation, the eyes were manual blinked 4–5 times to evenly spread the solution or hydrogel on the ocular surface. At each time point, 36 photos were taken using FlyCapture 2.7 (Point Gray Research, Inc., USA), and 5 were randomly selected for fluorescence signal quantification. Images were processed by ImageJ 1.84v. Area average gray‐scale signal of whole eye, including the cornea and conjunctiva were measured.

In this study, the hydrogel composed of 2.6 MDa HA‐VS (after dye conjugation) and HA‐SH of 8% DM, 1:1 mass ratio, and a total concentration at 0.45 mg/mL. Besides the hydrogel, the clearance behavior of two HA based formulation, fluorescently labeled 2.6 MDa HA‐VS at the same concentration as the hydrogel but without crosslinking (HA solution 1) and a viscous formulation containing 5 mg/mL fluorescently labeled 1500 kDa HA‐VS (HA solution 2) was also studied.

### 4.5 In vivo biocompatibility in laboratory animal

In vivo biocompatibility of the hydrogel was first evaluated on the ocular surface of three Sprague Dawley rats and two rabbits. The hydrogel composed of the 2.6 MDa HA‐VS and HA‐SH of 30% DM, 1:1 mass ratio, and a final concentration of 0.4 mg/mL. The eyes of rats were visually inspected before, immediately after and 24 h after hydrogel application for sign of inflammation or other visible damages. For the rabbits, the hydrogel was applied on the ocular surface for two times a day and visually inspected during gel application to one randomly chosen eye. The fellow eye was treated with saline as the control. Corneal staining by sodium fluorescein^45^ was performed for the rabbits immediately after instillation. Follow‐up examinations under anesthesia were also conducted after 1 and 2 weeks, each with one rabbit. After each time point, one rabbit was euthanized by pentobarbital rapid intravenous injection at marginal ear veins after examination. Treated eyes and control eyes were paraffin sectioned and stained with hematoxylin and eosin (H&E). Tissue integrity of corneal epithelium were observed under microscope. Gross morphology of the epithelium layer was compared.

### 4.6 Preparing material for veterinary canine study

The polymers were sterilely filtered through a 0.22 μm sterile filter, mixed to formulate as A3 in Table 2, and filled into a gamma radiated, 330 μL sterile monodose tube strip (Lameplast, Italy). The tube was sealed by a custom‐made ultrasonic tube sealer (Looker Machinery Co. Ltd., Guangzhou, China), and the sealed strips were incubated at 37°C for 24 h for gel formation and stored at 4°C. All procedures were performed in a laminar flow clean bench.

### 4.7 Biocompatibility in dog

Five healthy pet dogs were recruited from the Veterinary Specialty Hospital to evaluate the biocompatibility of the material with dogs. The hydrogel was instilled on the dog's eye twice a day, one in the morning and one in the evening for a course of 6 days. The amount of the eyedrop composition applied were varied, 250 μL for day 1–2, 200 μL for day 3–4, and 80 μL for day 5–6, respectively. A survey was conducted with the pet owners for subjective assessment of the tested eyedrop composition regimen.

### 4.8 Canine clinical trial of the hydrogel

This study was carried out in the Veterinary Specialty Hospital (VSH) in Hong Kong and was approved by the ethic committee of the hospital. All owners of the healthy subjects or patients with dry eye disease were given informed consent.

Dog patients that were diagnosed with dry eye syndrome and have been on 4–6 times a day artificial tear and two times a day cyclosporine treatment for over 1 year were enrolled in the study. Based on the clinical outcome of the previous cyclosporine treatment, patients were divided into two groups, cyclosporine responsive group and cyclosporine nonresponsive group depending on the Schirmer's test result when they were recruited. Dogs with grade 0 in the Schirmer's test were designated as cyclosporine responsive group, where grade 1–3 were designated as nonresponsive group. Both groups were switched to twice a day (BID) hydrogel application while maintaining the cyclosporine treatment. The cyclosporine was prepared at the pharmacy department of VSH. USP grade cyclosporine (Letco Medical, Hebei, China) was dissolved at 1% in USP grade corn oil and filled into a 10 mL sterile dropper bottle. Seven clinical signs including blephorospasm, conjunctiva hyperemia, discharge, Schirmer's test, corneal staining, keratitis and pigmentation were scored by the author (Derek Wai Yee Chow) at the initiation of the study and after 1‐month treatment with soft hydrogel. The grading scale is shown in Table 3.

## CONFLICT OF INTEREST

Yu Yu, Ying Chau, and Guanqun Zhou are employees and shareholders of Pleryon Therapeutics Ltd.

## AUTHOR CONTRIBUTIONS

**Yu Yu:** Conceptualization; data curation; formal analysis; funding acquisition; investigation; methodology; project administration; resources; software; supervision; validation; visualization; writing‐original draft; writing‐review & editing. **Derek Wai Yee Chow:** Investigation; methodology. **Laurence Chi Ming Lau:** Data curation. **Guanqun Zhou:** Data curation. **Woojin Back:** Data curation. **Jing Xu:** Data curation. **Sean Carim:** Data curation. **Ying Chau:** Conceptualization; funding acquisition; investigation; methodology; project administration; resources; supervision; writing‐original draft.

### PEER REVIEW

The peer review history for this article is available at https://publons.com/publon/10.1002/btm2.10227.

## Supporting information

**Movie S1**: Supporting Information

**Movie S2**: Supporting Information

**Movie S3**: Supporting Information

**Appendix S1**: Supporting Information

## Data Availability

The raw/processed data required to reproduce the above findings cannot be shared at this time due to time limitations.
