# Supplementary material for: A bioinspired synthetic soft hydrogel for the treatment of dry eye
Source: Bioeng Transl Med. 2021 Jun 5;6(3):e10227. doi: 10.1002/btm2.10227 (PMC8459603; doi:10.1002/btm2.10227)
Supplement: Supplementary file 4 — Appendix S1: Supporting Information [file BTM2-6-e10227-s001.docx]

**Supplementary Materials**

*Modification of of hyaluronic acid*

Hyaluronic acid was synthesized using the method developed in our lab (Figure S1) ^1,2^. Briefly, HA were dissolved in water and 5M NaOH was added drop wise to the polymer solution to a final concentration at 0.1M. Divinyl sulfone (DVS) were added instantly into the vigorously mixing polymers in excess to the hydroxyl groups of HA. For 120 kDa, the HA concentration was 20 mg/ml, DVS to OH ratio was 1.5x. For 670 kDa, the HA concentration was 10 mg/ml, DVS to OH ratio was 3x. For 2.6 MDa, the HA concentration was 2.5mg/ml, DVS to OH ratio was 6x. The relation between DM and reaction time under the above reaction condition is shown in Figure S2. Example NMR spectrums of HA-VS are shown in Figure S3. The polymers were purified by dialysis against deionized water. After dialysis, the polymers were stored as solution.

HA-SH was made by reacting HA-VS with excess dithiothreitol (DTT). The polymers were purified by dialysis against DI water adjusted to pH 4 using HCl. Because the HA-SH is made by converting all VS groups in HA-VS to SH group, its DM is controlled by controlling the DM of HA-VS. We only used Ellman’s Assay to determine the DM of HA-SH because the hydrogen atom of SH is not visible on NMR. Moreover, high MW HA-SH became insoluble in water (or D_2_O) after freeze drying, which resulted from the crosslinking of disulfide bond during freeze drying.


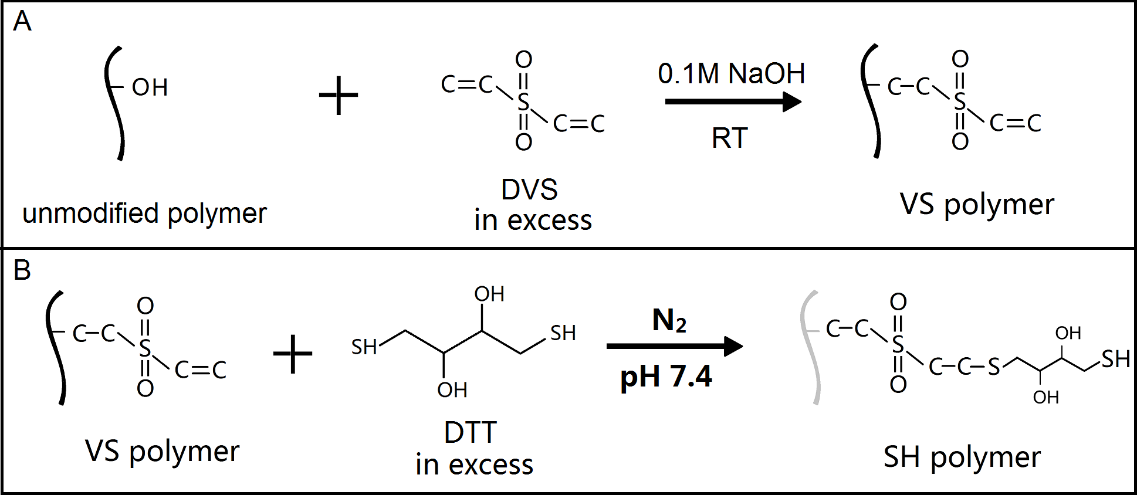


**Figure. S1** Chemical reaction scheme for functionalizing hyaluronic acid with A: vinylsulfone groups (VS) and B: thiol groups (SH)

**
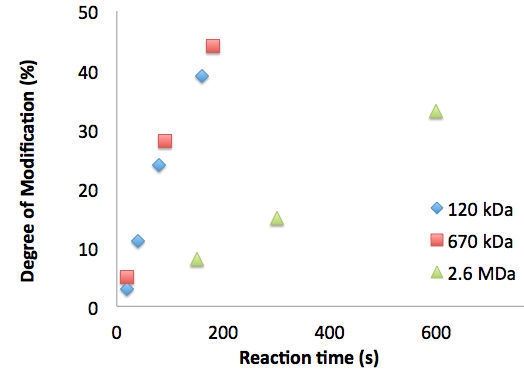
**

Figure S2. Controlling the degree of modification of polymers.


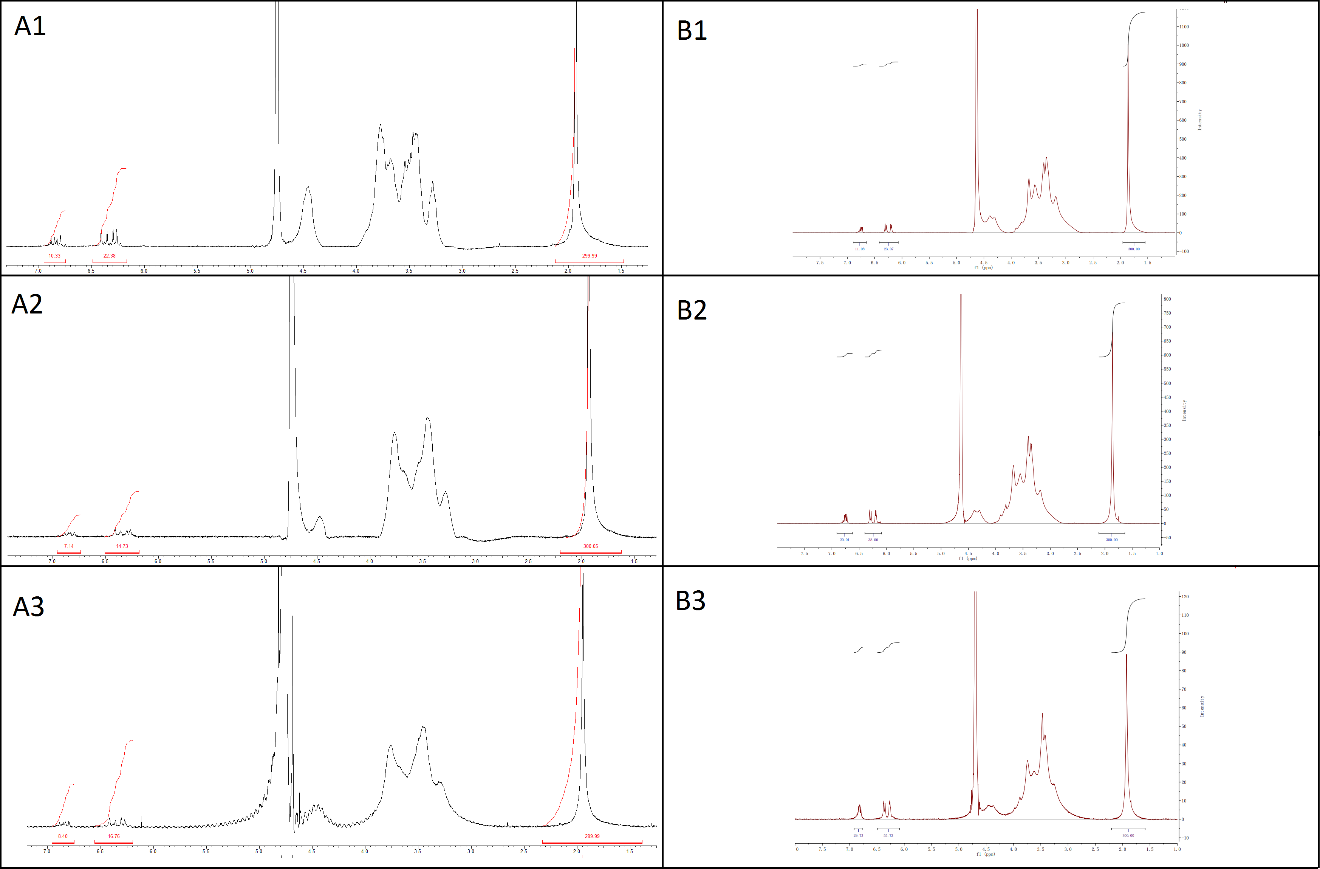


Figure S3: ^1^HNMR spectrum showing the successful modification of HA of A1: 120 KDa (10% DM), A2: 670 kDa (7% DM), A3: 2.6 MDa (9% DM) HA-VS. Examples showing the control of DM of 2.6 MDa HA of B1: 11.1%, B2: 20% and B3: 29.7%.

*Estimation of negative pressure at the puncta*

# Puncta is the small opening of the lacrimal system on the eye lid responsible for liquid drainage on the eye surface. The drainage is result from a negative pressure created from compression of the canaliculi during blinking[1–4]. The muscle of the eye lid is connected to a series of orbital muscles, which will squeeze the canaliculi during the eyelid closing phase and expand it as the opening phase during blinking. This squeezing cycle creates a negative pressure in the canaliculi to transport the precorneal fluid to the lacrimal duct [2,4,5].

# We first estimate the pressure at the puncta using Poiseuille’s law by simplifying the lacrimal canaliculi as a strait tube of small radius, neglecting the gravity, tube elasticity and the vertical canaliculi:

#

$$Q=\frac{\Delta P\pi R^{4}}{8\eta L}$$

# Where Q is the volumetric flow rate, ∆Ｐis the pressure drop in the tube, R is the radius, $\eta$ is the viscosity, L is the length (Fig. S9).

# Based on the clearance rate of the early 50% of a 25ul saline ($\eta$=1cP) on the ocular surface[6], Q was estimated to be 0.16 ul/s. The pressure at the end of canaliculi was estimated to be 0[2]. Based on the anatomical study of human cadaver, R and L of the lower canaliculi has been estimated to be about 0.5 mm and 10 mm accordingly[7].

# If we assume that only the lower canaliculus is responsible for the clearance[4], the pressure at the puncta would be about 0.07 Pa. If we assume both canaliculi drain the tear[8], the pressure may further be reduced to half (~ 0.04 Pa).

# The pressure in the canaliculi may increase in some occasional but commonly seen behavior including tightly closing the eyelid, rotating the eye ball and sneezing[9]. For The higher stress level of 0.2 Pa and 0.5 Pa may represent such condition.

*Biocompatibility of soft hydrogel*

Rabbit

In the rabbit study, single dose and multidose study were performed. A single dose of gel was applied on the cornea, ocular biocompatibility of gels was examined immediately afterwards by visual inspection for acute responses, and one week after experiment for chronic responses. The eyes were observed for changes in gross appearance and for any evidence of infection and discomfort, such as swelling, hyperemia, change in corneal clarity and mucoid discharge.

The cornea and lens remained clear with no indication of inflammation, and the animals did not display any signs of ocular discomfort neither immediately nor one week after the experiment. As HA gels took much longer time to be cleared than polymer solutions, we performed further biocompatibility examinations to verify the biocompatibility of the new gel formulations. The corneal staining was graded as zero (no staining on cornea or conjunctival）[10] for at 1 and 2 weeks post instillation, suggesting that HA gels did not cause any damage to the corneal epithelium. Histological examination of the rabbit cornea did not find any sign of inflammatory response not corneal epithelial damage (Fig. 3A and B of the main text).

To further evaluate the biocompatibility of the hydrogels, they were applied on rabbit eyes 3 times a day, for the course of 7 days and the eyes were observed visually (Fig. S3). No noticeable changes were observed in any of the rabbit eye.

*Fluorescent imaging system*

# All components except the lens were purchased from Edmund Optics Singapore Pte. Ltd. (Singapore). All lenses were donated by Newport Corp (U.S.A.). The excitation light was filtered by 467-498nm bandpass filter and the focused blue light illuminated the whole rabbit eye. The emission light was filtered by a 524-544nm bandpass filter and detected by a 1.3-megapixel monochrome CCD camera.

The system comprises an excitation module (comprising A, B, C and D) and an emission detection module (comprising E) were assembled. The excitation module comprises a light source (A), which illuminates full spectrum light with adjustable intensity levels (B), a fiber optic cable (C) for light transmission, a focusing lens, and a 467-498nm bandpass filter (D). The excitation module emits focused blue light illuminating the whole rabbit eye. The 467-498nm band of wavelengths covers the peak excitation wavelengths for FITC. The emission detection module (E) comprises a 524-544nm bandpass filter (which covers the emission wavelength of FITC).

# The monochrome CCD camera has a limited range for fluorescent signal detection. In order to maximize the detection range, images were acquired using at least 2 excitation intensities (one identical to the previous time point and one higher) when the emission intensity was low but still above the detection limit. This procedure could be used to extend the detection range because the emission intensity correlates linearly with dye concentration at all excitation intensity levels. In practice, experiments usually were started at illumination intensity level 4, this allowed the practitioner to follow the fluorescein signal until it diminished to about 1% of its initial fluorescent intensity. The detection limit for fluorescein was 0.01 nmol/ml at maximum illumination intensity. For each experiment, the fluorescent signal intensity at t=0 was defined as 100%.

# Before hydrogel or solution was instilled, the background fluorescence of each eye was measured at illumination intensity level 4, 5 and max. Appropriate illumination intensity was selected based on the fluorescent signal strength. For example, usually 0-min was imaged using illumination intensity 4. When the fluorescence signal was weak at 7-min, images would be recorded at illumination intensity level 4 and 5 respectively, and the 15-min images would be recorded at illumination intensity level 5 and max. The background intensity was subtracted accordingly for data analysis.

#
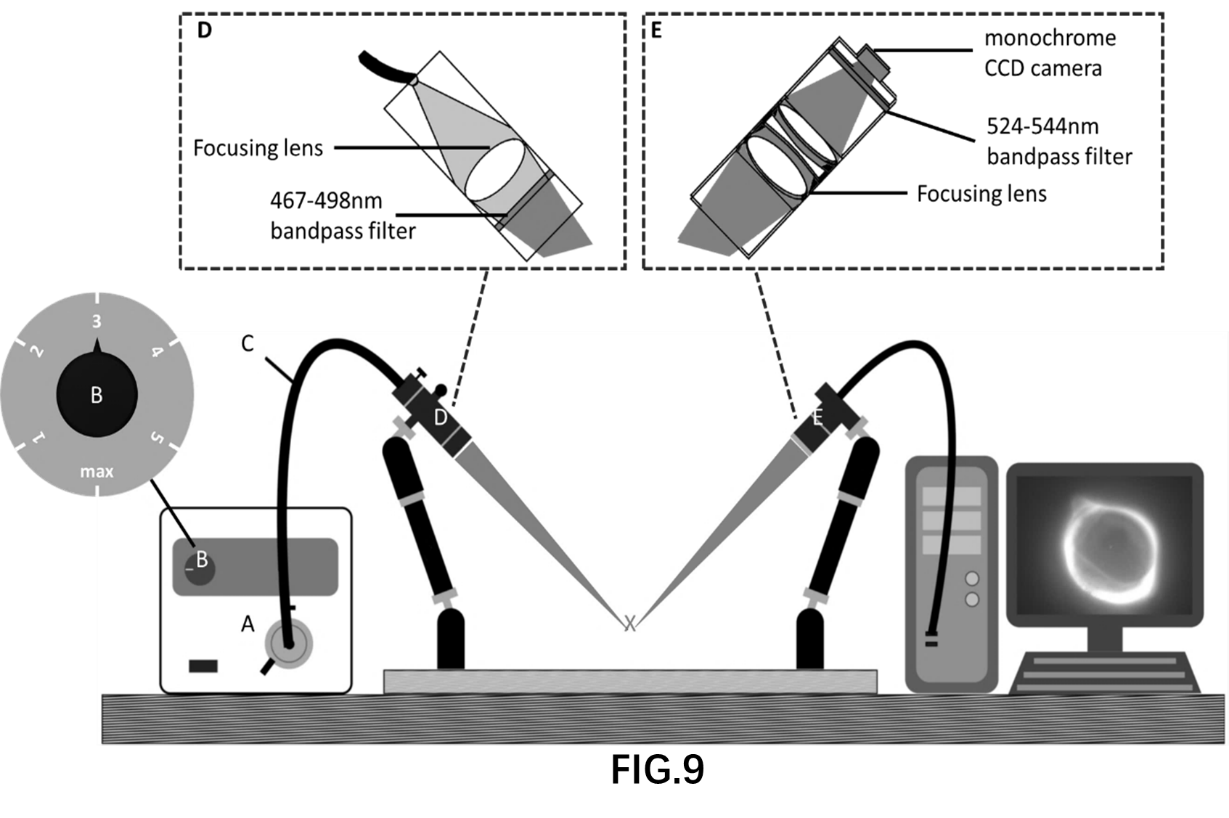


# Scheme S1: Schematic of the imaging system.

# ***Supplementary Figures***


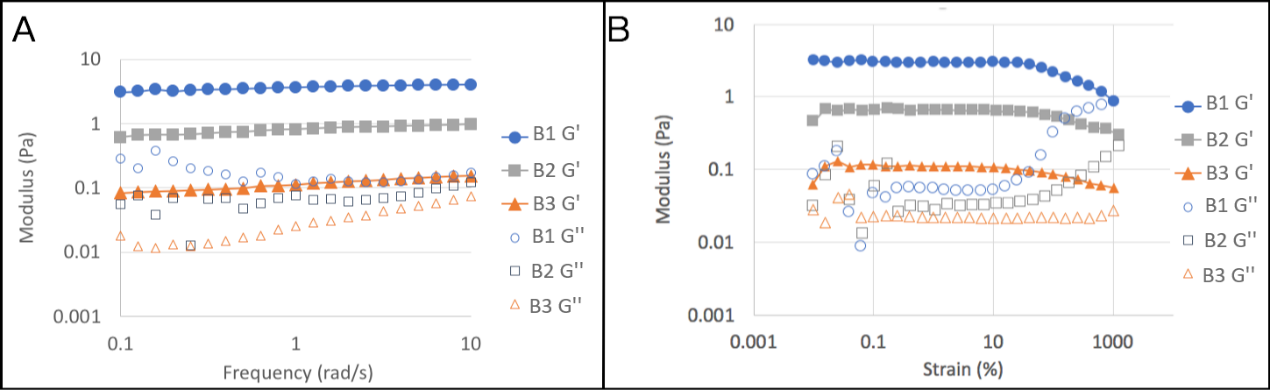


Figure S4.Frequency sweep and strain sweep test for three HA hydrogels composed of HA-VS and HA-SH of 2.6 MDa. The DM for both polymers were 10% and they were mixed at 1:1 ratio. The final concentration of polymer was 1.2 mg/ml, 0.8 mg/ml and 0.5 mg/ml for B1, B2 and B3 accordingly.

#
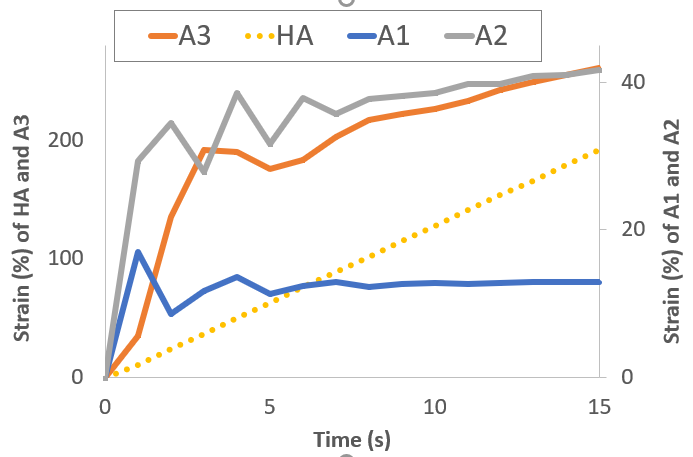


# Figure S5: Inertio-elastic ringing of the three hydrogel formulations (A1, A2 and A3) at 0.05Pa stress.

#
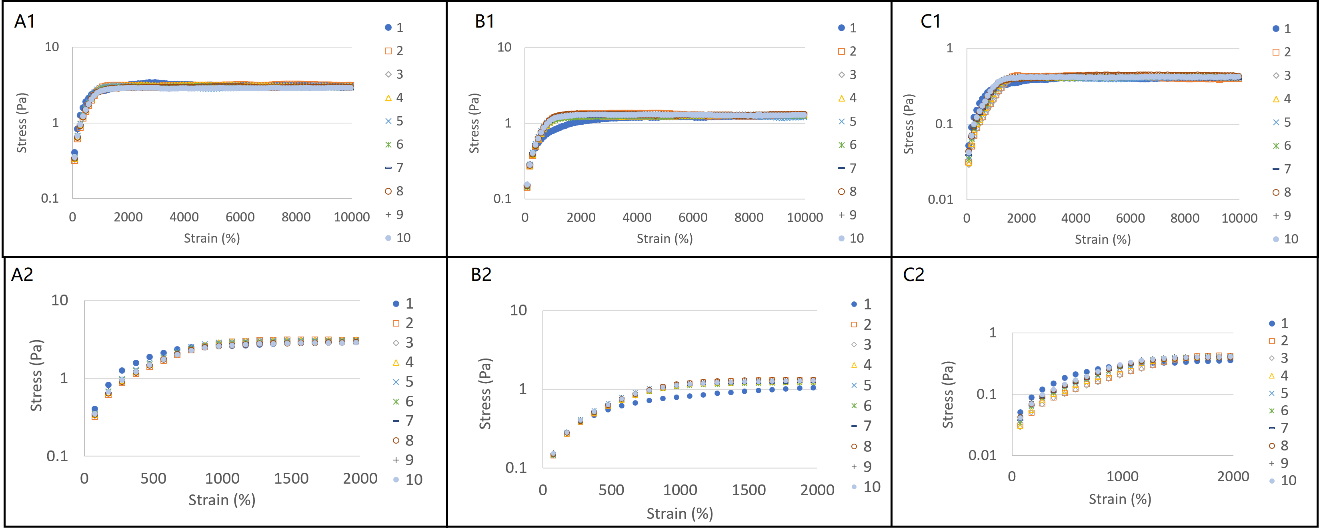


# Figure S6: The stress response to continuous shear test at 1/s shear rate for formulation A1 (Fig. S6 A1 and A2), formulation A2 (Fig. S6 B1 and B2), and formulation A3 (Fig. S6 C1 and C2). Fig. S6 A2, B2 and C2 are the initial 2000% deformation for Fig. S6 A1, B1 and C1.


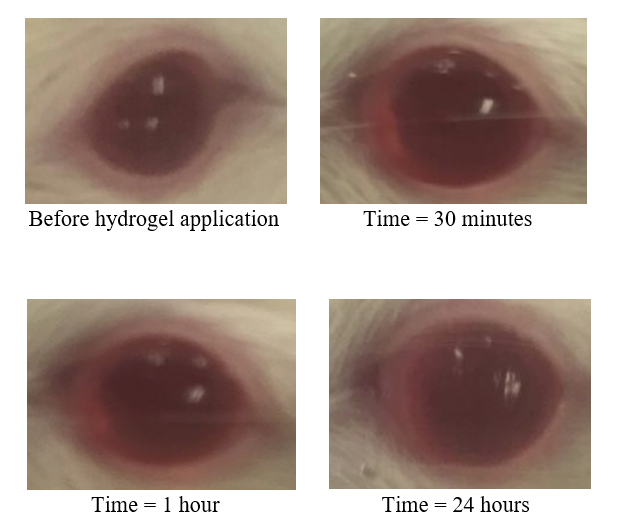

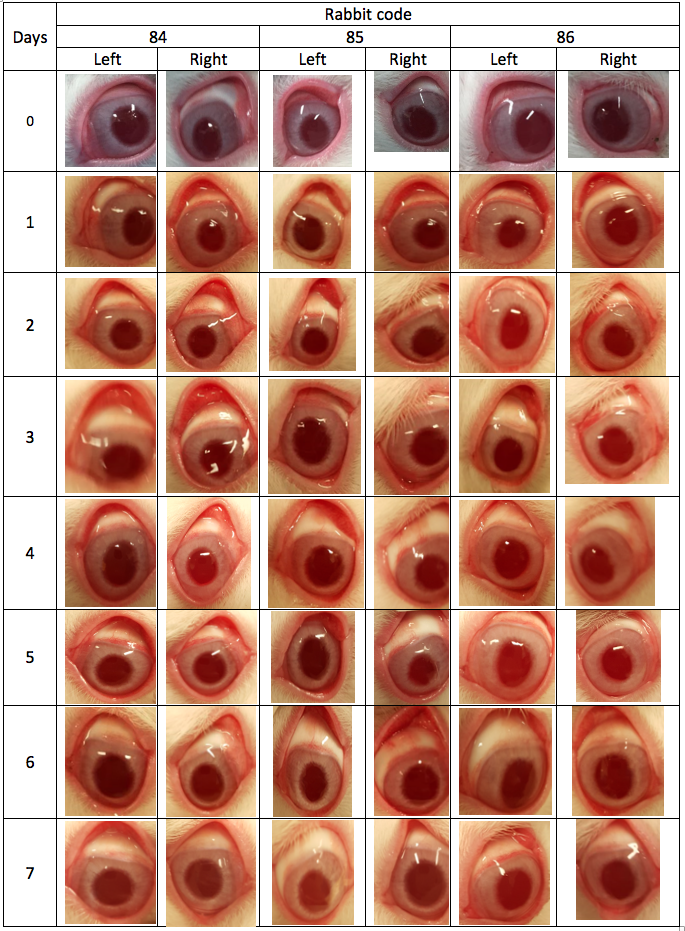


Figure S7. Photography of rat eyes before and after the application of hydrogels and the rabbit eyes before (day 0) and after hydrogel instillation at various days.

#
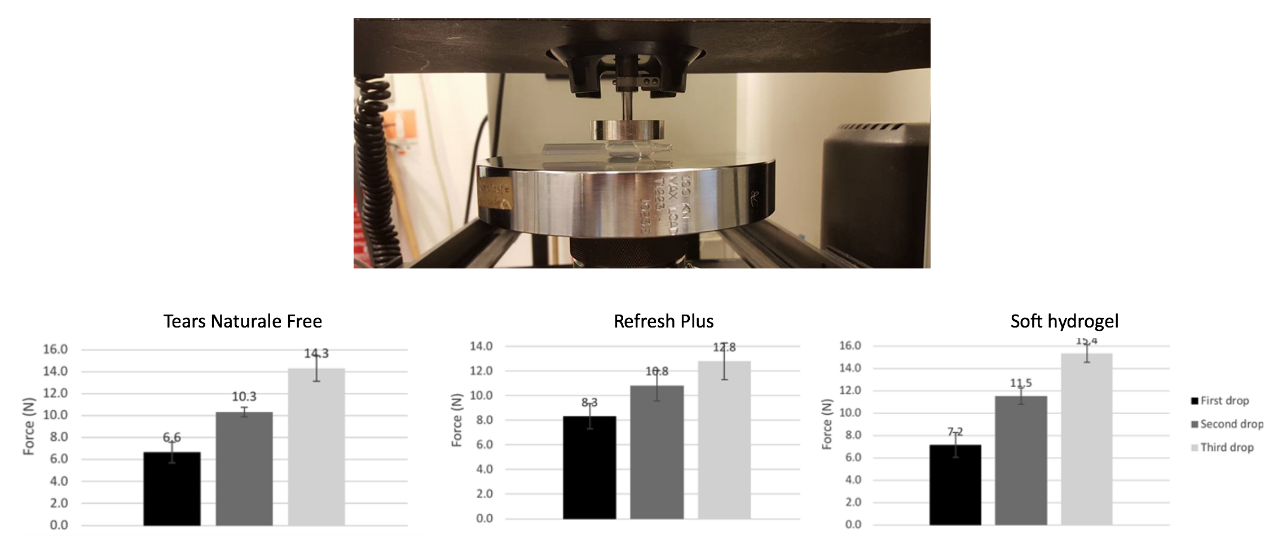


# Figure S8. Upper: the set-up of Instron for the measurement of squeezing force. Lower: force required to extrude the first, second the third drops of the material from the unidose eyedrop bottle. In this study, commercial artificial tears or the hydrogel forming polymer solution was loaded into a commercially available, empty 0.6ml blow-fill-seal (BFS) single use bottles (the original bottle from Tears Naturale Free). The hydrogel used in this study was made by mixing the HA-VS and HA-SH of 20% DM, at 1:1 mass ratio at a final concentration of 0.5mg/ml, and the DM of the HA-VS and HA-SH was about 20%. The compressive force required to squeeze the artificial tear or the hydrogel out of the bottle was measured by Instron 5567 H1540. Compression test has been performed with 100N load. The force was increased until the first drop was squeezed out of the bottle and at this time, the test was halted immediately. The force required to squeeze the second and the third drop out of respective bottles were performed in the same manner.

#
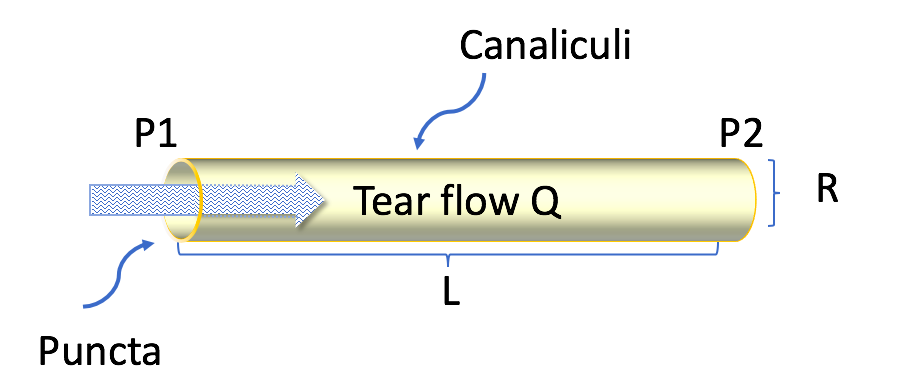


# Figure S9. A simplified schematics for the puncta and canaliculi.


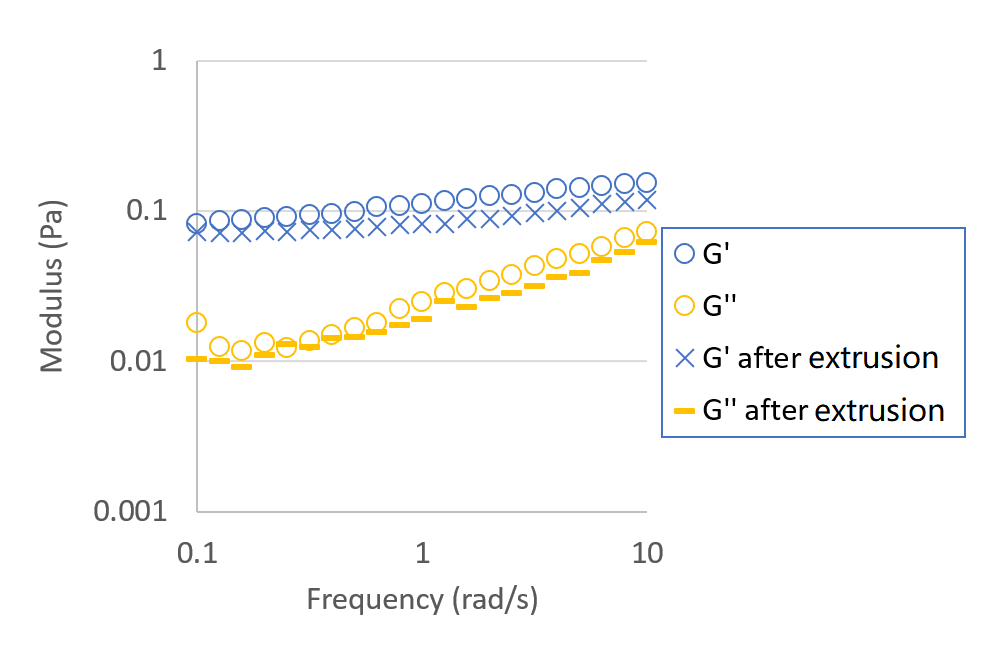


Figure S10: Frequency swept test for soft hydrogel before and after extrusion from an eye drop bottle. We tested this hypothesis in a worse-case scenario by extruding the hydrogel from a special multidose eye drop bottle (Pureflow 1500, Nemera, France). The nozzle of the bottle was specially designed such that it has several narrower channels for eye drops to travel from the bottle to the outside, and each drop size is only about 50 ul (compares to a total of > 1ml for the measurement). We found that the mechanical properties of soft hydrogels were almost identical before and after extrusion. A movie was recorded for the extrusion of soft hydrogel from the bottle (Movie S3).

# ***Supplementary Tables***

# Table S1: Radius of gyration of different polymers used commonly in eye drop formulation. The MW was set as 500 KDa for illustration purpose.

| Polymer | HA[14] | dextran[15] | CMC[16] | PEG[17] |
| --- | --- | --- | --- | --- |
| Rg (nm) | ~75 | ~20 | ~70 | 45 |

# ***Supplementary Movies***

# Movie S1: Physical appearance of soft hydrogel A2.

# Movie S2: Fluorescently labelled soft hydrogel added to water.

# Movie S3: Extrusion of soft hydrogel from an eye drop bottle (Pureflow 1500, Nemera, France).

Reference

[1] E.T. Detorakis, A. Zissimopoulos, K. Ioannakis, V.P. Kozobolis, Lacrimal outflow mechanisms and the role of scintigraphy: current trends., World J. Nucl. Med. 13 (2014) 16–21. https://doi.org/10.4103/1450-1147.138569.

[2] A.R.C. Çelebi, T.M. Önerci, Physiology of Lacrimal Drainage, in: Nasal Physiol. Pathophysiol. Nasal Disord., Springer Berlin Heidelberg, Berlin, Heidelberg, 2013: pp. 217–224. https://doi.org/10.1007/978-3-642-37250-6_16.

[3] N.C. Ahl, J.C. Hill, Horner’s muscle and the lacrimal system., Arch. Ophthalmol. (Chicago, Ill. 1960). 100 (1982) 488–93.

[4] H. Zhu, A. Chauhan, A mathematical model for tear drainage through the canaliculi, Curr. Eye Res. 30 (2005) 621–630. https://doi.org/10.1080/02713680590968628.

[5] M.G. Doane, Blinking and the Mechanics of the Lacrimal Drainage System, Ophthalmology. 88 (1981) 844–851. https://doi.org/10.1016/S0161-6420(81)34940-9.

[6] G.R. Snibson, J.L. Greaves, N.D. Soper, J.I. Prydal, C.G. Wilson, A.J. Bron, Precorneal residence times of sodium hyaluronate solutions studied by quantitative gamma scintigraphy., Eye (Lond). 4 ( Pt 4) (1990) 594–602. https://doi.org/10.1038/eye.1990.83.

[7] S.M. Tucker, J. V Linberg, L.L. Nguyen, A.J. Viti, W.J. Tucker, Measurement of the resistance to fluid flow within the lacrimal outflow system., Ophthalmology. 102 (1995) 1639–45.

[8] R.M. Rossomondo, W.H. Carlton, J.H. Trueblood, R.P. Thomas, A new method of evaluating lacrimal drainage., Arch. Ophthalmol. (Chicago, Ill. 1960). 88 (1972) 523–5.

[9] G. Wilson, R. Merrill, The lacrimal drainage system: pressure changes in the canaliculus., Am. J. Optom. Physiol. Opt. 53 (1976) 55–9.

[10] A.J. Bron, V.E. Evans, J.A. Smith, Grading of corneal and conjunctival staining in the context of other dry eye tests., Cornea. 22 (2003) 640–50.

[11] I. Teraoka, Polymer Solutions: An Introduction to Physical Properties, Wiley-Interscience, 2002.

[12] Y. Yu, Y. Chau, Formulation of in situ chemically cross-linked hydrogel depots for protein release: from the blob model perspective., Biomacromolecules. 16 (2015) 56–65. https://doi.org/10.1021/bm501063n.

[13] H.H. Bearat, B.H. Lee, B.L. Vernon, Comparison of properties between NIPAAm-based simultaneously physically and chemically gelling polymer systems for use in vivo., Acta Biomater. 8 (2012) 3629–42. https://doi.org/10.1016/j.actbio.2012.06.012.

[14] R. Mendichi, L. Soltés, A. Giacometti Schieroni, L. Šoltés, Evaluation of radius of gyration and intrinsic viscosity molar mass dependence and stiffness of hyaluronan., Biomacromolecules. 4 (2003) 1805–10. https://doi.org/10.1021/bm0342178.

[15] K.A. Granath, Solution properties of branched dextrans, J. Colloid Sci. 13 (1958) 308–328.

[16] A. Metodiev, Electric Properties of Carboxymethyl Cellulose, in: Cellul. - Fundam. Asp., InTech, 2013. https://doi.org/10.5772/56935.

[17] K. Devanand, J.C. Selser, Asymptotic behavior and long-range interactions in aqueous solutions of poly(ethylene oxide), Macromolecules. 24 (1991) 5943–5947. https://doi.org/10.1021/ma00022a008.
